# Radiomic analysis will add differential diagnostic value of benign and malignant pulmonary nodules: a hybrid imaging study based on [^18^F]FDG and [^18^F]FLT PET/CT

**DOI:** 10.1186/s13244-023-01530-6

**Published:** 2023-11-19

**Authors:** Jing Ning, Can Li, Peng Yu, Jingjing Cui, Xiaodan Xu, Yan Jia, Panli Zuo, Jiahe Tian, Lukas Kenner, Baixuan Xu

**Affiliations:** 1 Department of Nuclear Medicine, First Medical Center of Chinese PLA General Hospital, Beijing, China; 2 Christian Doppler Laboratory for Applied Metabolomics, Medical University of Vienna, Vienna, Austria; 3 Division of Nuclear Medicine, Department of Biomedical Imaging and Image-Guided Therapy, Vienna General Hospital, Vienna, Austria; 4 Department of Clinical Pathology, Vienna General Hospital, Vienna, Austria; 5 United Imaging Intelligence (Beijing) Co., Ltd., Beijing, China Yongteng North Road, Haidian District, Beijing, China; 6 Huiying Medical Technology Co., Ltd., Room C103, B2, Dongsheng Science and Technology Park, Haidian District, Beijing, China

**Keywords:** Pulmonary nodules, [^18^F]fluorodeoxyglucose PET, [^18^F]fluoropyrimidine PET, Radiomic analysis

## Abstract

**Purpose:**

To investigate the clinical value of radiomic analysis on [^18^F]FDG and [^18^F]FLT PET on the differentiation of [^18^F]FDG-avid benign and malignant pulmonary nodules (PNs).

**Methods:**

Data of 113 patients with inconclusive PNs based on preoperative [^18^F]FDG PET/CT who underwent additional [^18^F]FLT PET/CT scans within a week were retrospectively analyzed in the present study. Three methods of analysis including visual analysis, radiomic analysis based on [^18^F]FDG PET/CT images alone, and radiomic analysis based on dual-tracer PET/CT images were evaluated for differential diagnostic value of benign and malignant PNs.

**Results:**

A total of 678 radiomic features were extracted from volumes of interest (VOIs) of 123 PNs. Fourteen valuable features were thereafter selected. Based on a visual analysis of [^18^F]FDG PET/CT images, the diagnostic accuracy, sensitivity, and specificity were 61.6%, 90%, and 28.8%, respectively. For the test set, the area under the curve (AUC), sensitivity, and specificity of the radiomic models based on [^18^F]FDG PET/CT plus [^18^F]FLT signature were equal or better than radiomics based on [^18^F]FDG PET/CT only (0.838 vs 0.810, 0.778 vs 0.778, 0.750 vs 0.688, respectively).

**Conclusion:**

Radiomic analysis based on dual-tracer PET/CT images is clinically promising and feasible for the differentiation between benign and malignant PNs.

**Clinical relevance statement:**

Radiomic analysis will add differential diagnostic value of benign and malignant pulmonary nodules: a hybrid imaging study based on [^18^F]FDG and [^18^F]FLT PET/CT.

**Key points:**

• Radiomics brings new insights into the differentiation of benign and malignant pulmonary nodules beyond the naked eyes.

• Dual-tracer imaging shows the biological behaviors of cancerous cells from different aspects.

• Radiomics helps us get to the histological view in a non-invasive approach.

**Graphical Abstract:**

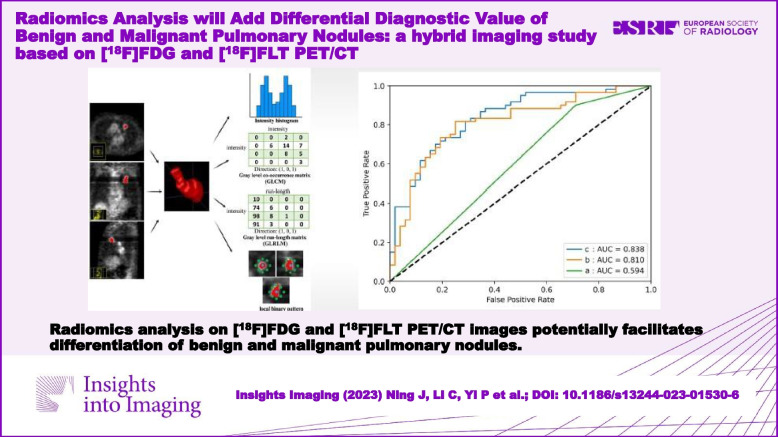

**Supplementary Information:**

The online version contains supplementary material available at 10.1186/s13244-023-01530-6.

## Introduction

Lung cancer is the leading cause of cancer-related deaths worldwide with a generally low survival rate [1, 2]. The increasing number of pulmonary nodules (PNs), a precancerous entity of lung cancer, can be detected and screened by computed tomography (CT) [3]. Malignant PNs detected at a late stage can increase mortality [4]. Therefore, it is crucial to improve the accuracy of early differential diagnosis between benign and malignant PNs.

In this scenario, [^18^F]fluorodeoxyglucose positron emission tomography ([^18^F]FDG PET), as a non-invasively practical imaging standard, is a key tool to detect lung cancer and evaluate its staging and prognosis [5]. The maximum standard uptake value (SUVmax), which is used clinically to assess tracer uptakes in lung cancer, provides information about the highest uptake point but not about tracer distribution within the tumor. Different features of the tumor such as cell proliferation, necrosis, microvessel density, blood flow, and hypoxia may be responsible for the heterogeneous distribution of FDG uptakes in different tumor types [6, 7]. Thus, [^18^F]FDG metabolic maps, showing the heterogeneous uptakes within the lesion, might be helpful in differentiating benign and malignant nodules [8]. Under this context, radiomic analysis, revealing a variety of quantitative radiomic features [9–11], could express the heterogeneity of a mass in a sequence of algorithms. Previous research demonstrated the benefit of radiomic analysis on [^18^F]FDG PET/CT images in the detection and identification of solitary pulmonary nodules (SPN) by distinguishing between benign and malignant PNs [12–14].

[^18^F]fluoropyrimidine ([^18^F]FLT) phosphorylated by thymidine kinase-1 (TK-1) can accumulate in cells during S-phase and is therefore a well-established tracer for monitoring cell proliferation [15, 16]. In general, [^18^F]FLT concentrates less in tumor cells compared to [^18^F]FDG, as S-phase is the only time for its accumulation [17]. In non-small cell lung cancer (NSCLC), intratumoral [^18^F]FLT uptake is directly correlated to Ki-67 expression which stains proliferating cells on histopathological slides [18, 19]. Since malignant lesions have a greater proliferation rate than benign mass, [^18^F]FLT may serve as a potent biomarker to distinguish malignant lesions from benign PNs [19–21].

In this study, we aim to discern the malignancy of pulmonary nodules by radiomic analysis based on [^18^F]FDG and [^18^F]FLT signatures and further compare its value with radiomic analysis based on [^18^F]FDG PET alone and visual analysis.

## Materials and methods

### Patients

The evaluation of retrospective data was approved in accordance with the ethical standards of the Chinese PLA General Hospital Committee.

From January 2016 to April 2018, we retrospectively reviewed data of 113 patients with inconclusive PNs on [^18^F]FDG PET/CT, all of whom underwent additional preoperative [^18^F]FLT PET/CT scans within a week [22]. An inconclusive PN was considered when the following criteria were met: (1) the lesions have apparently higher SUV than the rest of the lung; (2) there is no obvious evidence of nodal or distant metastasis, and (3) there are no definite indications of morphological malignancy, such as air bronchograms, spiculated or irregular margins, or lobulated shape.

The inclusion criteria of the study were (a) radiologically clear propensity to be diagnosed as a pulmonary nodule with a diameter of no more than 3 cm, (b) no definite diagnosis prior to [^18^F]FDG and [^18^F]FLT PET/CT examinations, (c) no treatments before PET/CT examination, (d) no indications of major organ dysfunctions or disorders, and (e) clear histopathologic identification or the endpoint of long-term follow-up.

### Imaging protocols

[^18^F]FDG and [^18^F]FLT were produced, and both their radiochemical purities are higher than 95%. Every patient fasted for over 4 h with a blood glucose level of < 11.1 mmol/L and rested in a quiet room for about half an hour. Then, the [^18^F]FDG tracer was given intravenously in a standardized dose of 3.70–4.44 MBq/kg. An hour after the tracer administration, every patient underwent [^18^F]FDG PET/CT scan (Discovery ST; GE Healthcare), and at least 1 day after [^18^F]FDG PET scan, the [^18^F]FLT tracer was also injected at a dose of 3.70–4.44 MBq/kg, and an hour later, every patient underwent [^18^F]FLT PET/CT scan (Discovery ST; GE Healthcare).

For both tracers’ scans, we ran the following settings to get low-dose CT (LDCT) scans to prevent patients from excessive radiation: 120 kV, 100–250 mAs with automatic adjustment, 0.8 s rotation, 1.25 mm collimation, and a pitch varied according to the geometry of CT detector (4, 8, or 16 slices). Meanwhile, PET was scanned in 2 min/bed, 3- or 4-bed positions (axial field view 15.7 cm) in three-dimensional mode with three iterations, and 21 subsets. Then, images were acquired. All the PET/CT data were reconstructed with the Fourier rebinding iterative algorithm and a Gaussian filter of 4 mm full width at half maximum.

### Visual analysis

Three clinicians with more than 10 years of diagnostic experience in pulmonary diseases conducted the visual analysis of PET/CT images. All the PET/CT image interpreters were blind to the patients’ information. The interpretation of PN malignancy was listed in Additional file 1. Also, the final diagnosis was determined based on all listed characteristics. Discrepancies between interpreters were resolved in a consensus meeting.

### Segmentation

All of the PET/CT data were analyzed by a semi-automated adaptive threshold method at the RadCloud platform (Huiying Medical Technology Co., Ltd., Beijing, China). Volumes of interest (VOIs) of the PN were initially drawn with a threshold of 40% of the SUVmax according to PET images via a commercial software (PET VCAR, GE Healthcare, Waukesha, WI, USA). After that, VOIs were checked visually on whether they have covered the whole components shown on the CT. If not, a lower threshold was then used [23]. If the VOI contains surrounding physical tissues, such as the adjacent myocardial activity, we would adjust its boundary manually [24]. All the segmentation was conducted by the same handler (a nuclear medicine physician with 4 years of experience in tumor drawing).

### Feature extraction

Both of the CT and PET images were analyzed, where radiomic feature calculations were performed within the same VOI in the settings of MATLAB (The MathWorks Inc.). When the respiratory motion leads to mismatches between CT and PET images, the extension of the VOI would be manually adapted to CT images. Before the computation of radiomic features, image voxel intensities were resampled into equally spaced bin widths of 0.1 [25]. The radiomic features extracted from [^18^F]FDG PET/CT images include shape features, histogram-based features, and texture features. Furthermore, texture features covered gray level cooccurrence matrix (GLCM), run length matrix (GLRLM), size zone matrix (GLSZM), and neighborhood gray-tone difference matrix (NGTDM) features. For more information about the description of the texture feature, see Additional file 1.

### Data analysis

To build the radiomic models, we took the histologically confirmed malignant or benign PN as the ground. In detail, firstly, the dataset was randomly divided into a training set and a test set in a ratio of 7:3, of which the training set was used for feature selection and modeling while the test set was used to test the models’ performance (the distribution of the dataset is shown in Table 1). Secondly, an analysis of variance (ANOVA) was applied to univariate feature selection to clarify the value of image features in the dataset in the differentiation of benign PNs from malignant. To be more detailed, features were eliminated if the *p* value exceeded 0.05. Then, the least absolute shrinkage and selection operator (LASSO) method was applied to the high-dimensional data regression to screen out the most valuable discriminative features from the training set [26], to prevent machine learning models from overfitting. The minimum mean square error (MSE) was calculated through fivefold cross-validation. Based on MSE under different parameters, the best penalty parameters and fitting model of LASSO were obtained. Finally, the non-zero coefficient features were selected for model training.

**Table 1:** The distribution of the dataset between benign and malignant PNs

	**Benign**	**Malignant**	**Dataset**
Training set	36	42	78
Test set	16	18	34
Total	52 (53.57%)	60 (46.43%)	112

### Development of an individualized prediction model

Two radiomics-based models were established using logistic regression (LR) for the distinguished diagnosis of benign PNs from malignant. The radiomic features selected by ANOVA and LASSO on [^18^F]FDG PET/CT images were used for modeling. Meanwhile, we classified PNs’ uptakes indicated on [^18^F]FLT PET images into no uptake, slight uptakes (lower than half the value of thoracic vertebrae), and apparent uptakes. In the model of FDG-based radiomic analysis, the threshold of the predictive probability value is 0.699. In the model of radiomic analysis based on FDG and FLT, the threshold of predictive probability value is 0.594. That is, if the value is more than the threshold, then the PN was regarded as malignant and otherwise it was benign. The classification was regarded as a signature, which was then integrated with the selected features of [^18^F]FDG PET/CT images to build another model. By doing so, we tried to find out whether the modeling performances could be improved. At last, the performances of the two models were tested with the 5-fold cross-validation method and then quantitatively assessed through the area under the curve (AUC), sensitivity, and specificity based on the receiver operating characteristic (ROC). Besides, a separate test set was run for verification.

## Results

A total of 123 lesions were recorded from 113 patients (74 men, 39 women) aged between 27 and 83 years (mean age 56.4 years), of which 63 benign lesions were respectively diagnosed as inflammation (*n* = 12), tuberculosis (*n* = 29), or other diseases confirmed by follow-up (*n* = 22) while 60 malignant lesions included adenocarcinoma (*n* = 30), squamous cell carcinoma (*n* = 16), small cell carcinoma (*n* = 1), bronchioloalveolar carcinoma (*n* = 6), and others (*n* = 7) (Table 2).

**Table 2:** The pathological diagnosis of 123 lesions

	**Pathological results**	**Number**
benign (*n* = 63)	Inflammation	12
	Tuberculosis	29
	Follow-up or others	22
Malignant (*n* = 60)	Adenocarcinoma	30
	Squamous cell carcinoma	16
	Small cell carcinoma	1
	Bronchioloalveolar carcinoma	6
	Follow-up or others	7

**Fig. 1:**
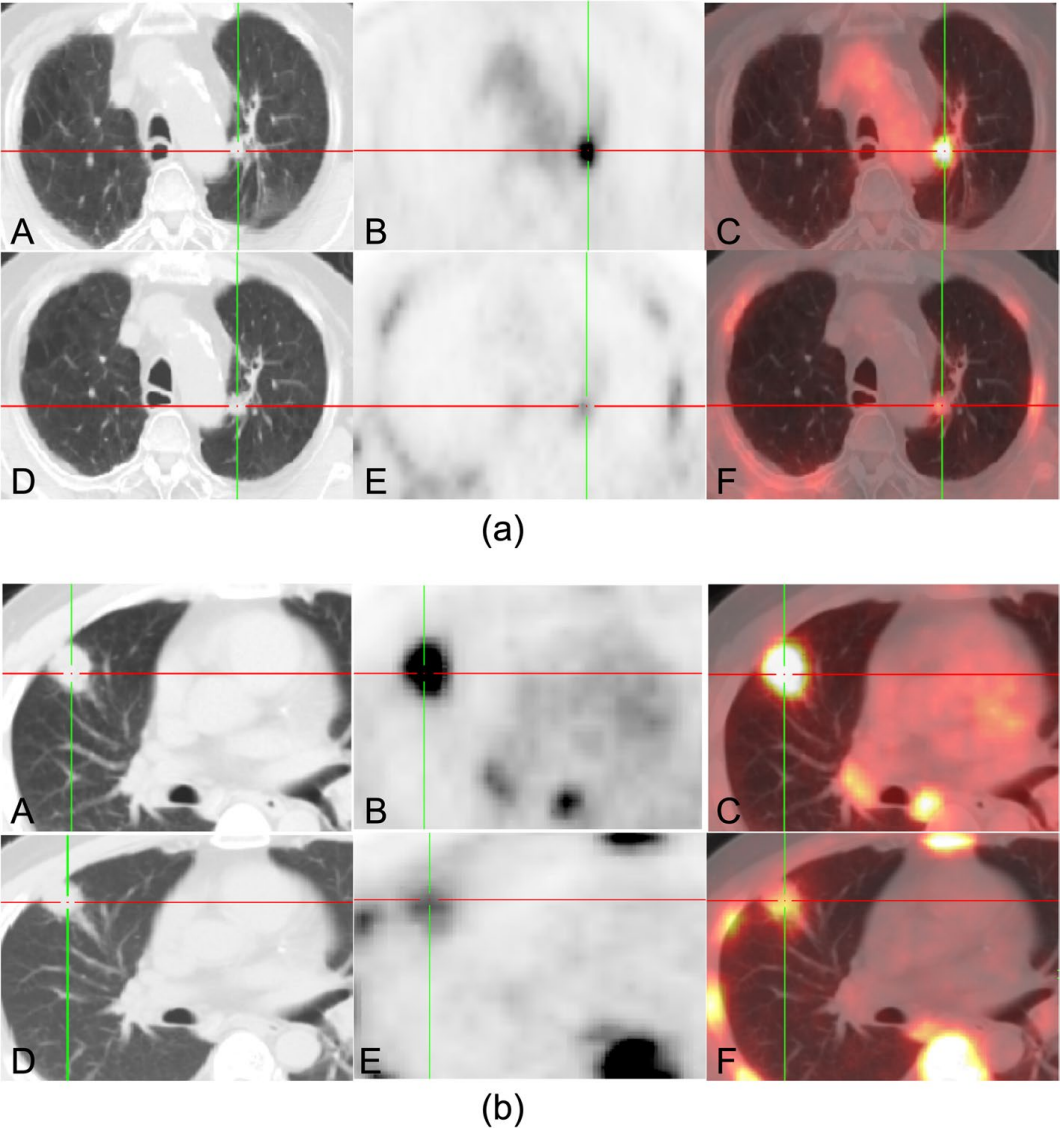
Representative dual-tracer PET/CT images of two cases. **a** Patient 1, male, 87 years old, malignant PN (lung cancer) (A–C). [^18^F]FDG PET/CT images show a small nodule without smooth, well-marginated borders, which lied near the aortic arch in the upper lobe of the left lung. The size was about 1.1 × 1.2 cm. Besides, higher glucose uptakes were observed within the nodule (SUVmax: 6.94) (D–F). [^18^F]FLT PET/CT images indicate higher tracer uptakes inside the nodule (SUVmax: 2.12). **b** Patient 2, male, 59 years old, benign PN (tuberculosis) (A–C). [^18^F]FDG PET/CT images show an irregularly shaped nodule with badly defined borders, which stood close to the pleura in the upper lobe of the right lung. The size was around 2.6 × 2.4 cm. Besides, there were tracer accumulation within the nodule (SUVmax: 8.26) (D–F). [^18^F]FLT PET/CT images indicated higher tracer uptakes inside the nodule (SUVmax: 2.48)

Based on the visual analysis of [^18^F]FDG PET/CT images, the diagnostic accuracy, sensitivity, and specificity were 61.6%, 90%, and 28.8%, respectively. Representative images of the two cases are shown in Fig. 1.

**Fig. 2:**
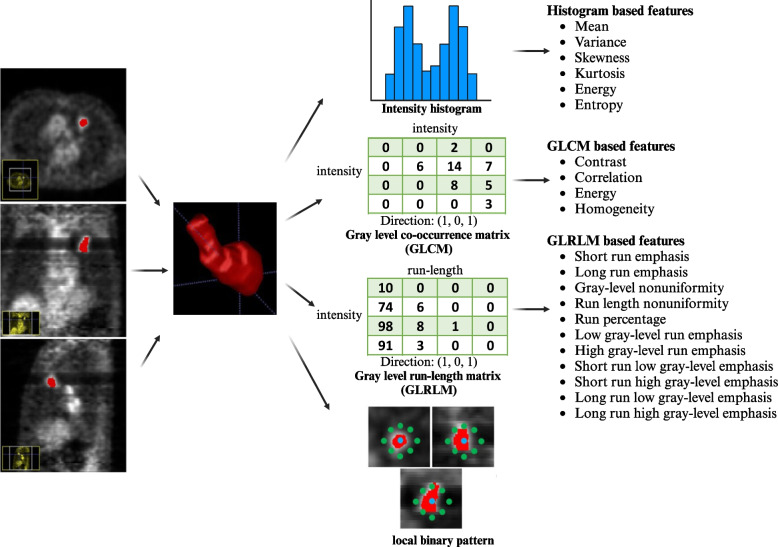
The workflows of VOI extraction and radiomic analysis. VOIs of the lesions were extracted based on its coronal, sagittal, and cross-sectional [^18^F]FDG and [^18^F]FLT PET/CT images. Then, the radiomic features were extracted according to VOIs

**Fig. 3:**
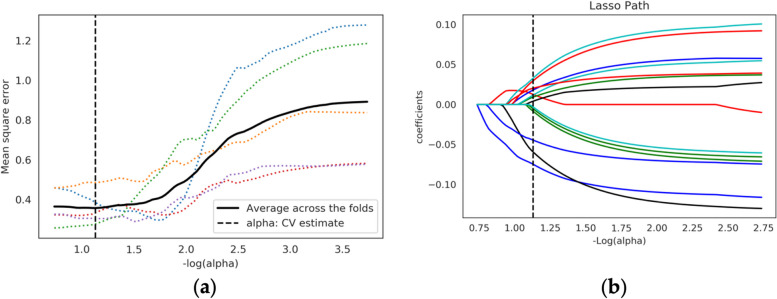
Feature selection using the LASSO binary logistic model. **a** The mean square error on each fold in fivefold cross-validation method. Vertical dotted line was drawn at the minimum mean square error of average. The optimal penalty parameter alpha was obtained based on the line. **b** LASSO coefficient solution path of the fourteen features. A coefficient profile plot was produced according to the log (alpha) sequence

**Fig. 4:**
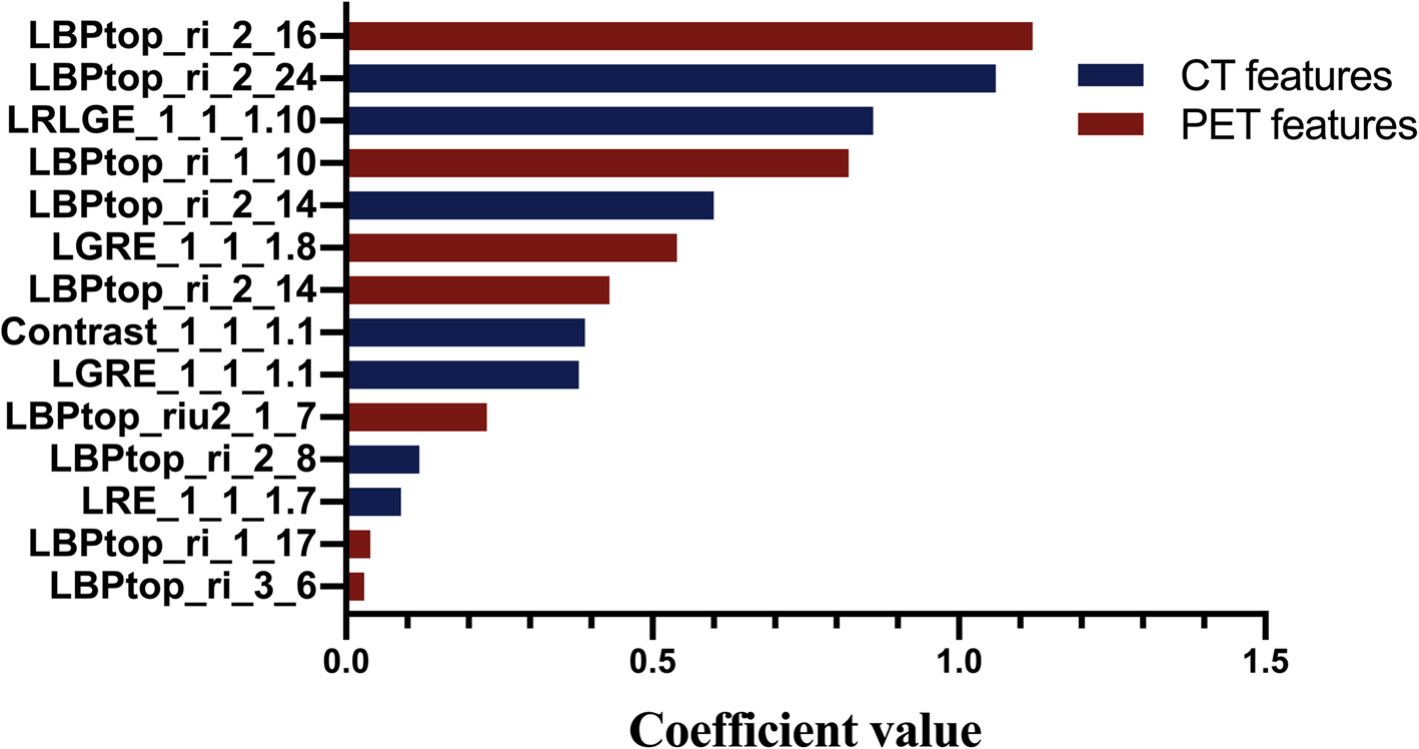
A total of 14 features were finally selected to predict the malignancy of PNs. These 14 features were ranked by the coefficient value of the model, among which there were 7 PET-based features and 7 CT-based features. The PET-based features were LBPtop_ri_2_16, LBPtop_ri_1_10, LGRE_1_1_1.8, LBPtop_ri_2_14, LBTtop_riu2_1_7, LBTtop_ri_1_17, and LBTtop_ri_3_6. The CT-based features were LBPtop_ri_2_24, LRLGE_1_1_1.10, LBPtop_ri_2_14, Contrast_1_1_1.1, LGRE_1_1_1.1, LBPtop_ri_2_8, and LRE_1_1_1.7

In total, 678 radiomic features were extracted from VOIs (shown in Fig. 2). After ANOVA preprocessing, 294 features were selected for subsequent LASSO analysis. The entire process is shown in Fig. 3. Finally, 14 features with the closest relation to the differentiation of benign and malignant PNs were selected based on *p* value. CT-derived features and PET-based features were ranked by the coefficient value of the model, which indicates the correlation level, and are categorized in Fig. 4. Among these 14 features, 7 features were derived from PET images and 7 features were CT-based. More details of radiomic features are shown in Additional file 2.

**Fig. 5:**
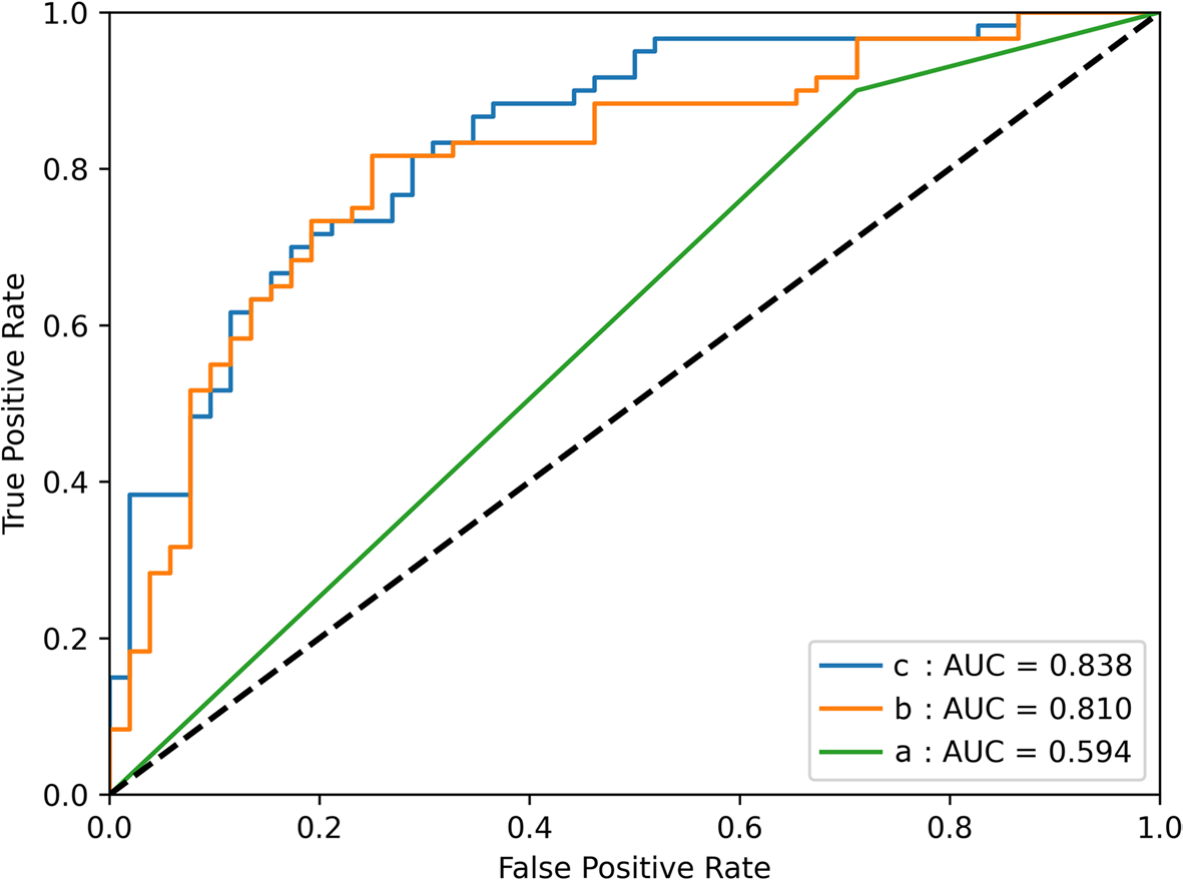
ROC curves for machine learning of radiomics. **a** ROC curves of visual analysis under the test set. **b** ROC analysis of radiomics based on [^18^F]FDG PET/CT under the test set. **c** ROC analysis of radiomics based on [^18^F]FDG PET/CT images plus [^18^F]FLT signature under the test set

We based the selected features and the features combined FLT signature to build two LR models, whose ROC are shown in Fig. 5. Based on [^18^F]FDG PET/CT plus [^18^F]FLT signature, the AUC, sensitivity, and specificity under the training set are 0.879, 0.810, and 0.750 in proper order while under the test set, they are 0.838, 0.778, and 0.750 successively. Based on [^18^F]FDG PET/CT images, the AUC, sensitivity, and specificity under the training set are 0.834, 0.786, and 0.778, respectively, while under the test set, they are 0.810, 0.778, and 0.688 in proper order. Table 3 reveals these results.

**Table 3:** The AUC, sensitivity, and specificity of three analysis methods under the test set

**Analysis methods**	**AUC**	**Sensitivity**	**Specificity**	**95% CI**
Radiomic analysis based on [^18^F]FDG and [^18^F]FLT PET/CT	0.838	0.778	0.750	0.765 to 0.912
Radiomic analysis based on [^18^F]FDG PET/CT alone	0.810	0.778	0.688	0.728 to 0.892
Visual analysis	0.594	0.900	0.288	0.521 to 0.667
Radiomic analysis based on [^18^F]FLT PET/CT	0.785	0.788	0.783	0.723 to 0.851

## Discussion

Early detection of lung cancer using LDCT, a clinically routine examination to discern the benign from malignant PNs, allows patients to receive timely treatment and better clinical outcomes, especially longer survival. Statistically, this could lead to a reduction of mortality in the long term. For accurate detection of lung cancer, the American College of Radiology (ACR) has developed the Lung Imaging Reporting and Data System (Lung-RADS) to standardize CT images [22, 27], which, however, still has its own limitations because LDCT decreased the sensitivity of PNs’ detection and further delayed the early identification of PNs’ malignancy. Therefore, it is extremely important to explore new methods to promote early and precise detection of PNs.

In this study, the performance of radiomic analysis supported by the combination of the dual tracers [^18^F]FLT and [^18^F]FDG PET/CT was shown to be better than [^18^F]FDG PET/CT, which convincingly demonstrated the added value of [^18^F]FLT PET/CT. Furthermore, the diagnostic efficacy of visual analysis tended to be lower than that of the radiomic analysis, which may be partly explained by the fact that the included subjects were difficult to diagnose on the basis of [^18^F]FDG PET/CT images. In particular, a large proportion of patients with tuberculosis toughened the problem because tuberculosis also presented high FDG uptakes on PET images, which makes it more difficult to differentiate from malignant PNs. However, machine learning-based radiomic analysis has the potential to distinguish solitary lung adenocarcinoma from tuberculosis [28]. Thus, it can be demonstrated that radiomic analysis was a viable and non-invasive potential tool for these cases, especially when combined with [^18^F]FLT PET/CT modality.

Generally speaking, the accuracy of image-reading results is subject to the interpretation criteria and physicians’ accumulation of professional skills and clinical experience. So, the precise identification of various diseases calls for the highest possible objective and quantitative view [29]. Although biopsy is thought of as the gold protocol for clear diagnosis of diseases, its limitations undoubtedly cannot be ignored, such as the invasivity, poor repeatability, higher incidence of secondary complications, and lack of whole body assessment or other spatial information other than puncture sites [30]. In both scenarios, radiomics is growing and thriving as a key area of clinical interest as a result of continued efforts to determine independent imaging features.

Radiomics-based analyses have been successfully used in assessing spatial patterns of non-uniform distribution in a way to measure the intra-lesion heterogeneity morphologically and quantitatively [7, 31]. Moreover, it has been reported that functional biomarkers including the glucose metabolism indicated by SUV maps do better than morphological parameters visualized on CT in the differentiation of PNs with different properties [32]. A previous study also indicated that integrating the morphological complexity and metabolic diversity of FDG improves the accuracy of lung cancer diagnosis, particularly by increasing the specificity [33]. These studies have shown that [^18^F]FDG PET/CT-based radiomic analysis, as a combined manner of both morphological complexity and FDG uptake heterogeneity, is of vital importance in facilitating accurate diagnosis and clear differentiation of diseases.

Van Velden et al. [33] observed lower FDG uptakes within malignant PN (lung cancer), which was considered as a reflection of metabolic heterogeneity within cancer lesions. Under the same circumstance, a quantitative parameter indicating FDG metabolic heterogeneity within tumors was put forward to evaluate NSCLC patients’ feedback to the clinical management [34]. To the same end, Tixier et al. [35] had clarified that heterogeneous distribution of FDG uptake within lesions could predict response to chemoradiation therapy in patients with esophageal cancer.

Currently, [^18^F]FLT has been recognized as a better-targeted tracer than [^18^F]FDG as it has a strong record of outstanding sensitivity in detecting primary carcinoma. However, clinical settings mainly focus on its potential to evaluate therapy response rather than other applications. Through the comparison between [^18^F]FDG and [^18^F]FLT uptakes in lung cancers, a conclusion was reached in a previous research that ^[18F]^FLT uptakes achieved extraordinarily high specificity in correlation with malignant tumors. Comparatively, [^18^F]FDG uptakes occurred in half the benign lesions, resulting in a non-negligible false-positive rate [36]. Also, Buck et al. detected [^18^F]FLT’s extreme insensitivity to lymph node staging (53%) [37]. However, no physiological concentration of the tracer was found in the brain, making it suitable for the interference-free diagnosis of brain metastases [37]. That was why he proposed [^18^F]FLT as a better tracer to assess the therapeutic feedback and clinical prognosis. Our findings with radiomic analysis based on [^18^F]FLT alone also indicated FLT performs better in the distinguish the benign PNs from malignant PNs. Similarly, a research enrolling 31 NSCLC patients demonstrated the sensitivity of [^18^F]FLT was much higher than that of [^18^F]FDG to primary lesions (74% vs 94%) (*p* = 0.003) [38]. Furthermore, a study of 18 subjects with lung nodules who underwent [^18^F]FLT and [^18^F]FDG scans summed up as a more favorable performance of dual-tracer imaging than one of tracers alone [21]. In this study, there was also a consistent conclusion that the [^18^F]FLT signature enhances the performance of radiomic analysis in distinguishing benign from malignant PNs.

From the statistical point of view, ANOVA and LASSO were used to screen the features in order to get rid of redundant features and avoid overfitting the models. Along the way of modeling, penalty was adopted as L_2_, which led to better learning of models and the two models constructed with satisfactory results. As pretreatment of feature selection, ANOVA ignored the correlation among features based on sparse assumption. So, the LASSO, a regression analysis method for variable selection and regularization, was adopted in order to further select features. Finally, we could assume that we have filtered out the most valuable features from a higher dimensional group of features.

This study has several limitations. There is some evidence suggesting that machine learning in FDG radiomic analysis is useful in distinguishing between subtypes with different levels of [^18^F]FDG uptake, such as squamous cell carcinoma and adenocarcinoma [39]. However, designed as retrospective, the present study had a relatively small sample size, which to some extent led to the heterogeneity in the lesions and selection bias in the analysis. Just as such, we did not analyze imaging biomarkers according to different histological subtypes of benign and malignant PNs. Furthermore, we only chose LDCT in the PET/CT scanner so as to protect subjects from excessive radiation exposure, unavoidably causing the absence of multiplanar reconstruction, contrast enhancement, or other classical methods. Although the effectiveness of LDCT has been confirmed, it may not be plausible enough to fully acclaim the diagnostic value of CT [40]. Additionally, the lack of motion correction on PET images could potentially lead to quantification errors and reduction of diagnostic confidence. At last, collective blind reading was conducted by professionals with various clinical backgrounds. That was how we tried to minimize the deviations from the right diagnosis. Yet, along with that, diverse window settings and perspectives may contribute to the discrepancy of judgment preferences. In other words, subjective factors cannot be completely excluded. Therefore, further research series with more homogeneous patients are needed to clarify the distinction between benign and malignant PN with a well-controlled and prospective design.

## Conclusions

Despite the clinical recognition of visual inspection, radiometric analysis has become more prevalent in the accurate differentiation of PNs by providing quantitative and comprehensive biological features. In particular, the addition of the [^18^F]FLT modality enriches the visualization of the heterogeneity of PNs under different aspects of cellular activity characteristics. Therefore, radiomic analysis based on PET/CT images with two/more tracers may be a clinical potential and a viable solution for the detectable evaluation of benign and malignant PNs, which requires further detailed exploration.

### Supplementary Information

**Additional file 1: Table S1.** The three categories of radiomic features on PET modality and CT modality. Notes: In the Column Feature Name, x_y_z represents a direction in 3D; d represents a cross-section and f represents the position in the histogram.

**Additional file 2.** The interpretation criteria of visual analysis.

## Data Availability

The datasets generated during and/or analyzed during the current study are available from the corresponding authors upon reasonable request.

## Abbreviations

[^18^F]FDG PET/CT: [^18^F]fluorodeoxyglucose positron emission tomography/computed tomography
[^18^F]FLT: [^18^F]fluoropyrimidine
LDCT: Low-dose CT
NSCLC: Non-small cell lung cancer
PN: Pulmonary nodule
SPN: Solitary pulmonary nodules
SUVmax: Maximum standard uptake value
TK-1: Thymidine kinase-1 TK-1
