# Supplementary material for: Radiomic analysis will add differential diagnostic value of benign and malignant pulmonary nodules: a hybrid imaging study based on [18F]FDG and [18F]FLT PET/CT
Source: Insights Imaging. 2023 Nov 19;14:197. doi: 10.1186/s13244-023-01530-6 (PMC10657912; doi:10.1186/s13244-023-01530-6)
Supplement: Supplementary file 1 — Additional file 1: Table S1. The three categories of radiomic features on PET modality and CT modality. Notes: In the Column Feature Name, x_y_z represents a direction in 3D; d represents a cross-section and f represents the position in the histogram. [file 13244_2023_1530_MOESM1_ESM.docx]

Radiomics Analysis will Add Differential Diagnostic Value of Benign and Malignant Pulmonary Nodules: a hybrid imaging study based on [^18^F]FDG and [^18^F]FLT PET/CT

**ELECTRONIC SUPPLEMENTARY MATERIAL**

**Supplementary Table 1.** The three categories of radiomics features on PET modality and CT modality.

| **Feature extraction methods** | **PET Feature No.** | **CT Feature No.** | **Feature name** |
| --- | --- | --- | --- |
| Intensity based features | 1-6 | 340-345 | Mean  Variance  Skewness  Kurtosis  Energy  Entropy |
| Gray level co-occurrence matrix based features | 7-58 | 346-397 | Contrast -> Contrast_x_y_z  Correlation -> Correlation_x_y_z  Energy -> Energy_x_y_z  Homogeneity -> Homogeneity_x_y_z |
| Gray level run-length matrix based features | 59-201 | 398-540 | Short run emphasis -> SRE_x_y_z  Long run emphasis -> LRE_x_y_z  Gray-level nonuniformity -> GLN_x_y_z  Run length nonuniformity -> RLN_x_y_z  Run percentage -> RP_x_y_z  Low gray-level run emphasis -> LGRE_x_y_z  High gray-level run emphasis -> HGRE_x_y_z  Short run low gray-level emphasis -> SRLGE_x_y_z  Short run high gray-level emphasis -> SRHGE_x_y_z  Long run low gray-level emphasis -> LRLGE_x_y_z  Long run high gray-level emphasis -> LRHGE_x_y_z |
| Rotation-invariant local binary pattern based features | 202-339 | 541-678 | LBPTOP_ri_d_f  Uniform rotation-invariant local binary pattern based features  LBPTOP_riu_d_f |

**Notes:** In the Column Feature Name, x_y_z represents a direction in 3D; d represents a cross-section and f represents the position in the histogram.
