# Supplementary material for: Radiomic analysis will add differential diagnostic value of benign and malignant pulmonary nodules: a hybrid imaging study based on [18F]FDG and [18F]FLT PET/CT
Source: Insights Imaging. 2023 Nov 19;14:197. doi: 10.1186/s13244-023-01530-6 (PMC10657912; doi:10.1186/s13244-023-01530-6)
Supplement: Supplementary file 2 — Additional file 2. The interpretation criteria of visual analysis. [file 13244_2023_1530_MOESM2_ESM.docx]

Radiomic Analysis will Add Differential Diagnostic Value of Benign and Malignant Pulmonary Nodules: a hybrid imaging study based on [^18^F]FDG and [^18^F]FLT PET/CT

**ELECTRONIC SUPPLEMENTARY MATERIAL**

**The interpretation criteria of visual analysis**

**Malignant pulmonary nodules on FDG PET/CT images:**

- Increased uptake of the radiotracer, indicating higher metabolic activity

- Larger size compared to benign nodules

- Irregular shape

- Indistinct or blurred margins, indicating invasive growth

- Evidence of surrounding lymph node involvement or distant metastasis

- Higher standardized uptake value (SUV) than benign nodules

**Benign pulmonary nodules on FDG PET/CT images:**

- Low or no uptake of the radiotracer, indicating low metabolic activity

- Smaller size compared to malignant nodules

- Round or oval shape

- Well-defined margins, indicating non-invasive growth

- Evidence of calcification, which can be seen on CT images

- Lower standardized uptake value (SUV) than malignant nodules
